# Environment-Related Genes Analysis of *Limosilactobacillus fermentum* Isolated from Food and Human Gut: Genetic Diversity and Adaption Evolution

**DOI:** 10.3390/foods11193135

**Published:** 2022-10-08

**Authors:** Yan Zhao, Leilei Yu, Fengwei Tian, Jianxin Zhao, Hao Zhang, Wei Chen, Yuzheng Xue, Qixiao Zhai

**Affiliations:** 1 Jiangsu Key Laboratory of Marine Bioresources and Environment, Jiangsu Key Laboratory of Marine Biotechnology, Jiangsu Ocean University, Lianyungang 222005, China; 2 Co-Innovation Center of Jiangsu Marine Bio-Industry Technology, Jiangsu Ocean University, Lianyungang 222005, China; 3 State Key Laboratory of Food Science and Technology, Jiangnan University, Wuxi 214122, China; 4 School of Food Science and Technology, Jiangnan University, Wuxi 214122, China; 5 National Engineering Research Center for Functional Food, Jiangnan University, Wuxi 214122, China; 6 Department of Gastroenterology, Affiliated Hospital of Jiangnan University, Wuxi 214122, China

**Keywords:** genomic analysis, *Limosilactobacillus fermentum*, source, adaptation, carbohydrate metabolism

## Abstract

*Limosilactobacillus fermentum* is ubiquitous in traditional fermented vegetables, meat products, and the human gut. It is regarded as a “generally recognized as safe” organism by the US Food and Drug Administration. So far, the genetic features and evolutionary strategies of *L. fermentum* from the human gut and food remain unknown. In this study, comparative genomic analysis of 224 *L. fermentum* strains isolated from food and human gut (164 *L. fermentum* strains isolated from human gut was sequenced in our lab) was performed to access genetic diversity and explore genomic features associated with environment. A total of 20,505 gene families were contained by 224 *L. fermentum* strains and these strains separated mainly into six clades in phylogenetic tree connected with their origin. Food source *L. fermentum* strains carried more carbohydrate active enzyme genes (belonging to glycosyltransferase family 2, glycoside hydrolase family 43_11, and glycoside hydrolase family 68) compared with that of human gut and *L. fermentum* derived from food showed higher ability to degrade xylulose and ribose. Moreover, the number of genes encoding otr(A), tetA(46), lmrB, poxtA, and efrB were more abundant in food source *L. fermentum*, which was consistent with the number of CRISPR spacers and prophages in *L. fermentum* of food source. This study provides new insight into the adaption of *L. fermentum* to the food and intestinal tract of humans, suggesting that the genomic evolution of *L. fermentum* was to some extent driven by environmental stress.

## 1. Introduction

*Limosilactobacillus fermentum* is a facultatively anaerobic, gas-producing, and obligately heterofermentative bacterium [1]. In 1901, some basic physiological and biochemical characteristics (cellular morphology, nutritional requirement, and carbohydrate fermentation) of this strain were described for the first time in Bergey’s Manual of Systematic Bacteriology; it can actively ferment sugars, such as glucose, fructose, sucrose, lactose, mannose, and ribose, but usually shows little or no fermentation of xylose, cellobiose, and trehalose [2]. *L. fermentum* can convert the carbohydrates in food to acid to alter flavor, prolong shelf life, and improve nutritional quality. It is used as a common starter culture in traditional fermentation of fruits and vegetables [3,4]. Furthermore, it was regarded as a “generally recognized as safe” organism by the US Food and Drug Administration in 2013. Accumulating evidence has shown that *L. fermentum* is ubiquitous in the intestinal tract of humans [5]. In 2017, Duar synthesized the habitat and phylogenetic analysis of *Lactobacillus* and speculated that *L. fermentum* could have experienced a change in host-adapted lifestyle (existing in invertebrate or vertebrate hosts) to a free-living lifestyle (mainly isolated from the environment and plant) [6].

Phenotypic and biochemical characteristics were the main criteria for the differentiation of *L. fermentum* in the next few decades after it was discovered; it was reclassified and differentiated from *L. reuteri* and *L. cellobiosus* in 1980 and 2004, respectively [7]. Molecular techniques involving randomly amplified polymorphic DNA, amplified 16S rDNA restriction analysis, and ribotyping were developed in the 1990s and have been applied to identify *Lactobacillus* at the species level [8]. *L. fermentum* belonged to the genus *Lactobacillus* and it was reclassified as a new genus *Limosilactobacillus* in 2020 [9]. *L. fermentum* can now be directly identified without culturing based on the bacterial 16S ribosomal RNA amplicon with advances in high-throughput DNA sequencing [10]. The rapid development in DNA sequencing from the late 20th century to the 21st century has made the whole-genome sequencing of *L. fermentum* possible, proving to be both time- and cost-saving [11]. Since 2008, more and more genomes of *L. fermentum* strains have become accessible from the National Coalition Building Institute database [12]. The genetic diversity of *L. fermentum* strains isolated from different geographical areas or food-types was performed by Tong Dan et al. using phylogenetic analysis based on multilocus sequence typing [13]. Our previous research also showed that *L. fermentum* strains isolated from human fecal samples clustered into three distinct clades in the phylogenetic tree [12]. Duar et al. analyzed the evolution and natural history of nearly 200 *Lactobacillus* spp. and *Pediococcus* spp. and showed that *Lactobacillus* genus with the same ecological niche clustered together in the phylogenetic tree, which provided valuable information for the industrial and therapeutic applications of certain genera [6]. Qing Li and Michael also showed that vertebrate-adapted lactobacilli (*L. reuteri*, *L. delbrueckii*, *L. salivarius,* and so on) harbored some special characteristics (tetracycline resistence, degrade sucrose to glucans and fructans, acid resistance, and so on) compared with insect-adapted lactobacilli [14].

Research shows that *L. fermentum* is commonly used in food and biotechnology. For example, Zhang et al. show that *L. fermentum* accounts for 22.6% of strains in traditional fermented yak milk and can produce bacteriocin-like substance to inhibit the growth of spoilage microorganisms in milk [15]. Fei Huang et al. show that *L. fermentum* can ferment longan polysaccharides and produce arabinose, galactose, rhamnose, and mannose and thus improve solubility and reduce the viscosity and particle size of longan pulp [16]. Xue Zhang et al. find that Harbin red sausage fermented with *L. fermentum* instead of nitrite has more free amino acids and these strains are alternative for producing pink, cured color through converting Mb(Fe^3+^) to cured meat pigment NO-Mb(Fe^2+^) [17]. Irene Falasconi et al. indicate that *L. fermentum* can be used as starter cultures for sourdough since they can produce exopolysaccharide, CO_2_, amylase, and form acidification [18]. In addition, *L. fermentum* has also been shown to ferment vegetable [19] and fruit juice [20,21] and can increase the total phenolic and total flavonoid contents, prevent spoilage, and improve the shelf life of food. The physiological characteristics of lactobacilli, including the structure of exopolysaccharides [22] and lactic acid production [23], have been shown to directly influence the texture and flavor of fermented foods. Rodolphe et al. also showed that clustered regularly interspaced short palindromic repeat (CRISPR)-based technologies could be applied to alter some genes of food microbiota to control spoilage bacteria and pathogens and improve the taste and sensory properties of food products [24].

Evidence has also shown that *L. fermentum* can act as a probiotic and provide health benefits in humans and animals. Luciana et al. showed that the health-promoting properties of *L. fermentum* in cancer and neurodegenerative and metabolic disorders may be due to their antioxidant properties [25]. *L. fermentum* strains have shown significant potential as a therapeutic tool for pathogenic infection [26], colitis [27], cardiovascular disease [28], and hepatic injury [29]. Products such as cheese, yogurt, beverages, capsules, and pills containing *L. fermentum* strains (including *L. fermentum* CECT5716, PCC, and ME-3) have begun to dominate the global probiotics market [7]. Researchers have indicated that the metabolites (exopolysaccharides [30], antimicrobial compounds [31], bile salt hydrolase [32], organic acid, and lactase [33]) produced by probiotics in the host tissue may modulate host biology and disease processes. Genes related to the synthesis of bile salt hydrolase [32], branched short-chain fatty acids [34], reuterin, and cobalamin [35] have been clarified.

Since probiotic properties and fermentation characteristics of *L. fermentum* are intimately linked to specific genes in the strain, it is essential to understand the genomic traits of *L. fermentum* strains. Furthermore, the nutrition characteristics, temperature, pH, oxygen, osmotic pressure, and redox potential [34] of human gut and fermentation food could affect the evolutionary change of *L. fermentum* and it is harbored in both environments [35]; whether this difference would cause the loss or occurrence of specific genes is still unknown. In this study, comprehensive genomic analysis of 224 *L. fermentum* strains derived from the human gut and food (164 were isolated from the gut of humans in our lab and 60 were obtained online) were collected, and the genes encoding orthologous proteins, antibiotic resistance, carbohydrate-active enzymes, CRISPR/Cas9, virulence factors, and prophage in *L. fermentum* were analyzed.

## 2. Materials and Methods

### 2.1. Genome Sequence

A total of 164 *L. fermentum* strains were isolated from 153 healthy Chinese human gut (samples WX111-WX115 were from the same person, samples HN112, HN14-HN1110 were from the same person) and Fast DNA Spin Kit was used to extract the DNA of *L. fermentum* strains [36]. Then, the DNA amplicons were sequenced using the Illumina Hiseq 10 platform (San Diego, CA, USA). Sixty *L. fermentum* genomes were obtained from the NCBI microbial genome database. The basic information of these strains is provided in Table S1.

### 2.2. Average Nucleotide Identity Values, Pan-, and Core-Genome, and Phylogenetic Analyses

Average nucleotide identity (ANI) was calculated using Python and pan and core genomes were analyzed using PGAP 1.2.1 [37]. OrthoMCL1.4 was used to analyze orthologous genes, and the maximum likelihood method was used to perform a phylogenetic analysis of 224 *L. fermentum* strains (based on 615 orthologous genes).

### 2.3. Clusters of Orthologous Groups (COGs) Analysis

The genomes of 224 *L. fermentum* strains were uploaded to BLAST against all annotated Clusters of Orthologous Groups (COGs) in the COG database (https://www.ncbi.nlm.nih.gov/COG (accessed on 5 June 2021)). The dominant COGs in each clade are shown in Table S2.

### 2.4. Carbohydrate Metabolism

The genomes of 224 *L. fermentum* strains were uploaded to BLAST against all annotated CAZyme proteins in the Carbohydrate-Active enZyme (CAZy) database. These genomes were also uploaded to BLAST and annotated against sequences in the non-redundant protein sequence database (NR), and the enzymes involved in carbohydrate metabolism were analyzed.

### 2.5. Antibiotic Resistance Genes (ARGs)

Antibiotic resistance genes (ARGs) of 224 *L. fermentum* strains were annotated using the Comprehensive Antibiotic Research Database (CARD) (http://arpcard.mcmaster.ca (accessed on 6 July 2021)).

### 2.6. CRISPR-Cas Systems

CRISPR loci in *L. fermentum* strains were characterized using CRISPRFinder (https://crisprcas.i2bc.paris-saclay.fr/CrisprCasFinder/Index (accessed on 6 July 2021)).

### 2.7. Prophage Identification

Prophage prediction of *L. fermentum* strains relied on similarity searches against a database of prophage genes (http://phaster.ca/ (accessed on 9 August 2021)).

### 2.8. Statistical Analysis

PERMANOVA and pairwise comparison analysis was used to analyze the difference between groups (* *p* < 0.05, ** *p* < 0.01, and *** *p* < 0.001). The data of the ANI, pan and core genomes, COGs analysis, carbohydrate metabolism, ARGs, CRISPR-Cas systems, and prophage identification were visualized using R (ggplot2 package). Microsoft PowerPoint and Adobe Illustrator were used to visualize and assemble the pictures.

### 2.9. Data Deposition

The genomes of 164 *L. fermentum* strains screened in our lab were sequenced and uploaded to the Sequence Read Archive database in NCBI Data Bank with biosample accession numbers SAMN15891013-SAMN15891179.

## 3. Results

### 3.1. Genetic Diversity and Phylogenetic Analysis of 224 L. fermentum Strains

**Figure 1:**
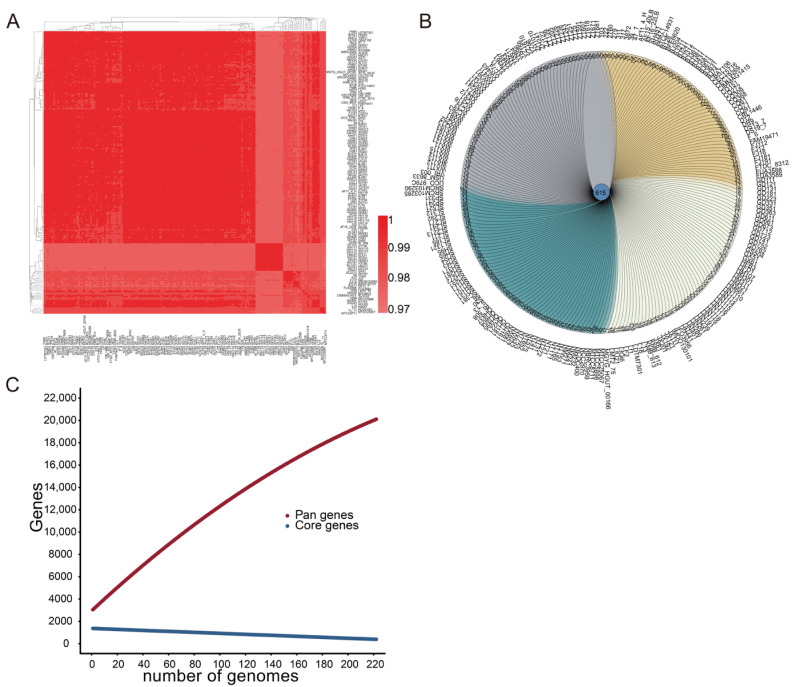
The genomic and genetic characteristics of 224 *L. fermentum*. (**A**) Average nucleotide identity scores of 224 *L. fermentum*. The color gradation from light red to dark red indicates an increase in genome similarity; (**B**) Venn diagram of the homologous clusters of 224 *L. fermentum*; (**C**) Pan- and core-genome of 224 *L. fermentum*. The abscissa axis represents the number of genomes of *L. fermentum* and the vertical axis represents the number of pan- and core-genomes.

The nucleotide-level genomic similarity between the coding regions of every two genomes of *L. fermentum* strains in this study was greater than 97% (Figure 1A). The similarities, differences, and relationships between the genomes of 224 *L. fermentum* strains are presented in the Venn diagram in Figure 1B; 615 genes were shared by the genomes of all *L. fermentum*, and 11–525 unique genes were present in each strain. Pan-genome analysis revealed that the number of pan genes increased sharply as the genome of *L. fermentum* strains increased, and 20,505 gene families existed in the genomes of 224 *L. fermentum* strains. Compared with the pan-genome curve, the core-genome curve decreased flatly and 502 core genes were shared by the genomes of 224 *L. fermentum* strains (Figure 1C).

**Figure 2:**
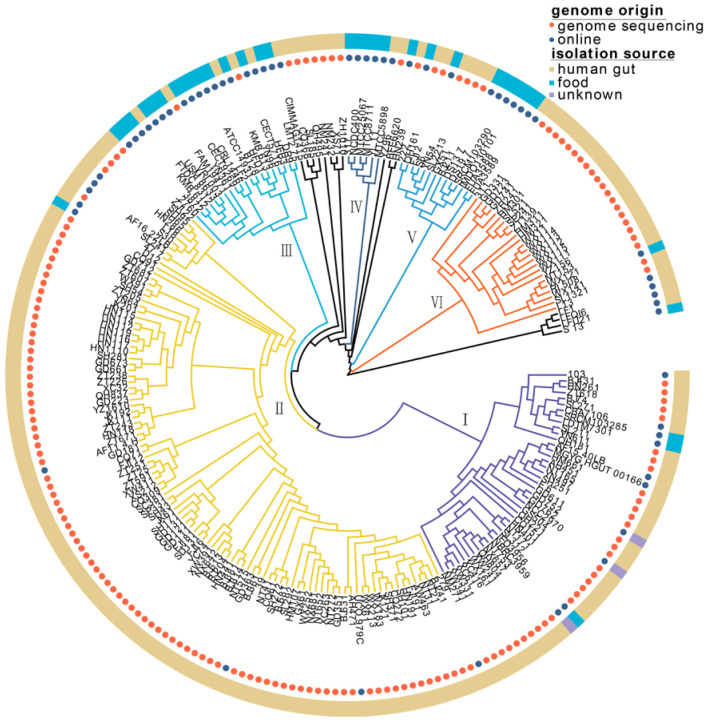
Phylogenetic analysis of 224 *L. fermentum* isolated from human intestinal tract and food based on 615 orthologous genes (All strains mainly clustered into phylogenetic clade I, II, III, IV, V, and VI). The genome origin is annotated with orange and blue circles and the isolation source of *L. fermentum* strains is notated with yellow, blue, and purple stripes.

On the phylogenetic tree, 224 *L. fermentum* strains were divided into six clades (clades Ⅰ, Ⅱ, Ⅲ, Ⅳ, Ⅴ, and Ⅵ) (Figure 2). Of the 60 *L. fermentum* strains obtained from NCBI, 22 *L. fermentum* strains were derived from human fecal samples and 35 *L. fermentum* strains were isolated from food sources (Table S1). *L. fermentum* strains belonging to clades Ⅲ and Ⅳ mostly originated from food sources, while *L. fermentum* strains isolated from the human gut mainly clustered in clades Ⅰ, Ⅱ, and Ⅵ. Clade Ⅴ included *L. fermentum* strains with half human and half food sources.

### 3.2. Analysis of Clusters of Orthologous Groups (COGs) in L. fermentum Strains

**Figure 3:**
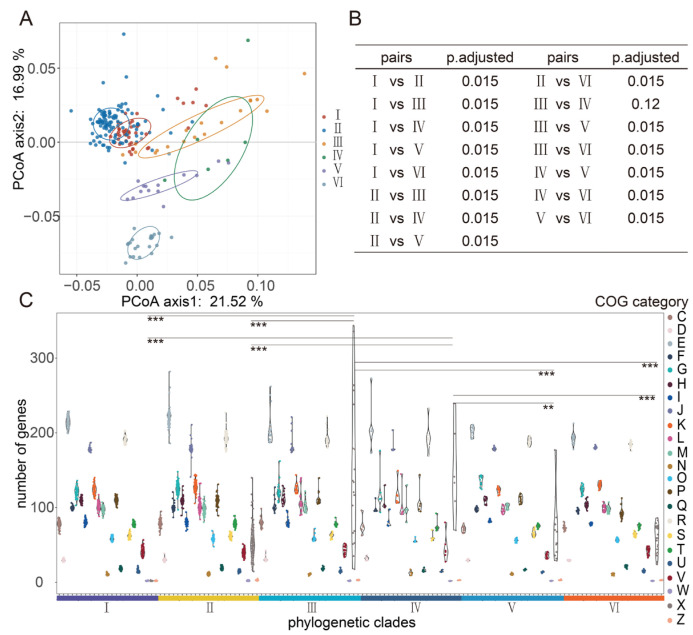
Clusters of orthologous groups (COGs) in the genome *L. fermentum* strains distributed in phylogenetic clades Ⅰ, Ⅱ, Ⅲ, Ⅳ, Ⅴ, and Ⅵ separately. (**A**) Principal coordinates analysis (PCoA) of COGs in the genome of *L. fermentum* strains in 6 main phylogenetic clades; (**B**) PERMANOVA and pairwise comparison analysis COGs in the genome of *L. fermentum* strains in 6 phylogenetic clades; (**C**) the violin plots show the number of genes annotated with diverse COG functional categories in *L. fermentum* in the phylogenetic clades Ⅰ, Ⅱ, Ⅲ, Ⅳ, Ⅴ, and Ⅵ and * indicate a significant difference in functional categories X between different group (** *p* < 0.01, *** *p* < 0.001).

A total of 1434 clusters of orthologous groups were harbored by the genome of 224 *L. fermentum* strains in the COG database. Principal coordinates analysis (PCoA) of COG between six phylogenetic clades showed that orthologous groups of proteins in the genomes of clades Ⅰ and Ⅱ were more similar and proteins in clade Ⅵ were differentiated from those of any other groups (Figure 3A). PERMANOVA and pairwise comparison results showed no significant difference between clades Ⅲ and Ⅳ (Figure 3B). Among all COG functional categories, genes categorized as mobilome, prophages, and transposons (functional categories of X in COGs) varied the most between different clades, and these genes in clades Ⅲ and Ⅳ were significantly higher than those in any other clade (Figure 3C).

Analysis of functional categories enriched in *L. fermentum* genome may provide new ideas for identifying the environmental characteristics or stress. Based on the above results, we observed significant differences in *L. fermentum* genomes of human and food source. Then, LEfSe analysis of COG categories in two groups of *L. fermentum* genomes was performed and the result showed that the number of dominant COG functional categories belonging to human source *L. fermentum* and food source *L. fermentum* were 31 and 74, respectively. Food source *L. fermentum* strains were relatively lower than that of human source and they contained significantly low dominant COG categories (Table S2). Remarkably, some functional genes belonging to COG category of mobilome, prophages, and transposons were widely shared by *L. fermentum* strains of food source. Among these, genes annotated as COG2826, COG3328, COG2801, COG0675, COG1943, COG2963, COG3464, COG3436, and COG3293 were all related to transposase and were most differentially distributed in the food source *L. fermentum* genomes. Compared to food source, human gut source *L. fermentum* genomes had significantly more genes annotated as energy production and conversion, amino acid transport, and metabolism. Dominant COG categories sorted by LDA (linear discriminant analysis) score greater that 2.5 were COG1309 (DNA-binding protein), COG1028 (NAD(P)-dependent dehydrogenase), COG0538 (isocitrate dehydrogenase), COG0531 (serine transporter YbeC), COG0716 (Flavodoxin), and COG1063 (threonine dehydrogenase or related Zn-dependent dehydrogenase). Overall, compared to food source *L. fermentum* strains, human source *L. fermentum* genomes contained significantly more dominant COG categories, such as functional categories of C, E, G, K, L, and R.

### 3.3. Identification of Carbohydrate Metabolism in L. fermentum Strains

**Figure 4:**
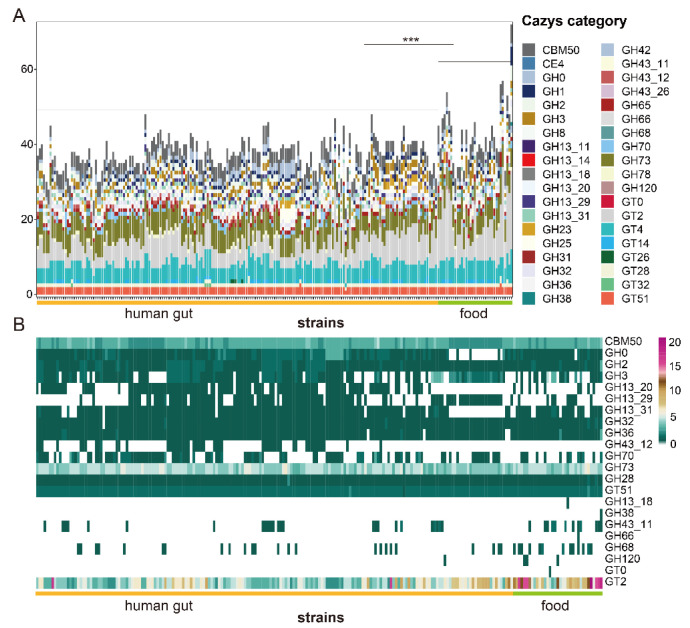
Genes encoding carbohydrate-active enzymes (CAZy) in the genome of *L. fermentum* strains isolated from human gut and food. (**A**) Stacked bar chart of carbohydrate-active enzyme (CAZy) categories in the genome of *L. fermentum* derived from different sources and * indicate a significant difference in the number of CAZys between different group (*** *p* < 0.001); (**B**) heatmap of the number of specific CAZy categories in the genome of *L. fermentum* strains from human gut and food. (The items of CAZy category with LDA score greater than 2 using linear discriminant analysis effect size analysis are listed).

CAZyme families included in the genomes of *L. fermentum* strains were glycoside hydrolases (GHs), glycosyltransferases (GTs), carbohydrate esterases (CEs), and carbohydrate-binding modules (CBMs). Among these, glycosyltransferase family 2, glycosyltransferase family 4, glycoside hydrolase family 73, and carbohydrate-binding module family 50 were major families in genomes of both two groups of *L. fermentum* strains. CAZyme families of food source and human feces source *L. fermentum strains* were comparative analyzed by PERMANOVA and pairwise comparison and result showed that CAZyme genes in group of food source were significantly higher than that of human gut source. A relatively large number of glycoside hydrolases not yet assigned to a family (GH0) were included in human feces source *L. fermentum strains* (Figure 4A). In order to find out whether or not there is a statistically significant difference between two *L. fermentum* groups, LEfSe analysis with a Kruskal–Wallis test was used. Of 38 CAZyme families, 22 CAZyme families were significantly different between the human sources and food sources. The number of glycoside hydrolase family 3, glycoside hydrolase family 13_20, and glycoside hydrolase family 13_29 was common in human-derived *L. fermentum*, but they were rare in food-derived strains (Figure 4B), while glycosyltransferase family 2, glycoside hydrolase family 43_11 and glycoside hydrolase family 68 were dominant in “food source” *L. fermentum strains*.

**Figure 5:**
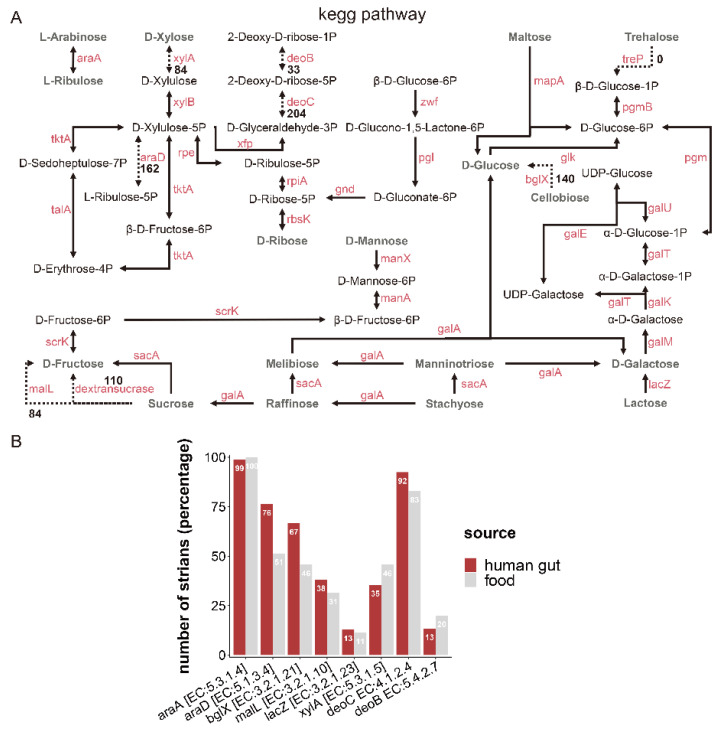
Metabolism of carbohydrates and related metabolic pathways of *L. fermentum*. (**A**) Schematic representation of CAZy in the metabolic pathway of the 224 *L. fermentum* strains. The solid lines indicate that more than 220 *L. fermentum* strains contained the enzyme. The dotted lines meant that the enzyme was present in fewer than 220 *L. fermentum* strains and the number of strains containing the enzyme is denoted by the dotted line; (**B**) number of genes encoding the enzymes (L-arabinose isomerase [EC: 5.3.1.4], araA; L-ribulose-5-phosphate 4-epimerase [EC: 5.1.3.4], araD; beta-glucosidase [EC: 3.2.1.21], bglX; oligo-1,6-glucosidase [EC: 3.2.1.10], malL; beta-galactosidase [EC: 3.2.1.23], lacZ; xylose isomerase [EC: 5.3.1.5], XylA; deoxyribose-phosphate aldolasebeta-glucosidase [EC: 4.1.2.4], deoC; phosphopentomutase [EC: 5.4.2.7], deoB) in the genome of *L. fermentum* strains from different sources.

Based on the non-redundant protein sequence database (NR), the enzymes involved in carbohydrate metabolic pathways are presented in Figure 5A. Related genes encoding enzymes involved in the metabolism of L-arabinose, D-galactose, D-glucose, D-ribose, D-mannose, maltose, melibiose, manninotriose, sucrose, stachyose, lactose, and raffinose were present in almost all *L. fermentum* strains (more than 220). Genes encoding enzymes (xylose isomerase [EC: 5.3.1.5], XylA; beta-glucosidase [EC: 3.2.1.21], bglX; dextransucrase [EC: 2.4.1.5], and oligo-1,6-glucosidase [EC: 3.2.1.10]) involved in the metabolism of D-xylose, cellobiose, and sucrose were strain-specific and their existence was unrelated to the isolation source of *L. fermentum* strains. L-ribulose-5-phosphate 4-epimerase [EC: 5.1.3.4] was ubiquitous in the genome of *L. fermentum* strains from different sources (Figure 5B). For example, the abundance of genes encoding AraD [EC: 5.1.3.4] and bglX [EC: 3.2.1.21] was significantly higher in *L. fermentum* of human source than food source, while the coverage of genes involving XylA and deoB [EC: 5.4.2.7] in food-derived *L. fermentum* was higher than that of human-derived.

### 3.4. Characteristic of Antibiotic Resistance Genes in L. fermentum Strains

**Figure 6:**
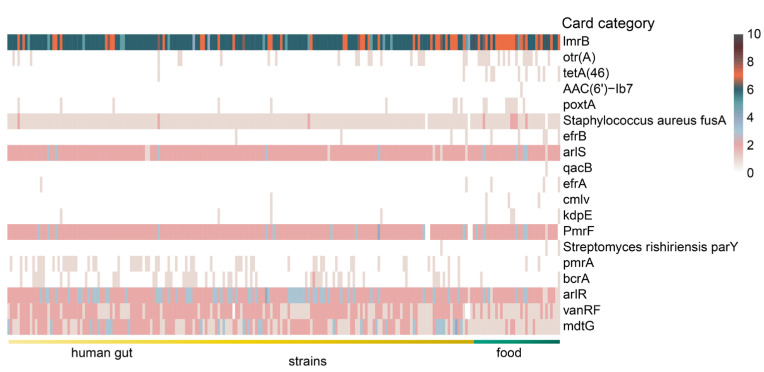
Heatmap of the number of differential antibiotic resistance genes annotated with the comprehensive antibiotic resistance database in the genome of *L. fermentum* from human gut and food. (The items of antibiotic resistance gene with LDA score greater than 2 using linear discriminant analysis effect size analysis are listed).

The genomes of 224 *L. fermentum* strains were annotated using the Comprehensive Antibiotic Resistance Database (CARD) and a total of 58 antibiotic resistance gene categories were found in the genomes of 224 *L. fermentum* strains. Based on LEfSe analysis with a Kruskal–Wallis test, 19 significantly different antibiotic resistance gene families were shown in Figure 6. Of note, antibiotic resistance gene family *otr(A)* (tetracycline antibiotic) and *tetA(46)* (tetracycline antibiotic) were almost exclusively found in food source *L. fermentum* strains. Number of genes belonging to card category *lmrB* (lincosamide antibiotic), *poxtA* (tetracycline antibiotic, phenicol antibiotic, and oxazolidinone antibiotic), and *efrB* (fluoroquinolone antibiotic, rifamycin antibiotic, macrolide antibiotic) were also dominant in *L. fermentum* strains in food. For human-derived *L. fermentum* strains, antibiotic resistance gene family *pmrA* (fluoroquinolone antibiotic), bcrA (peptide antibiotic), *arlR* (fluoroquinolone antibiotic), *vanRF* (glycopeptide antibiotic), and *mdtG* (phosphonic acid antibiotic) were all more abundant.

### 3.5. Identification of CRISPR-Cas Systems in L. fermentum Strains

**Figure 7:**
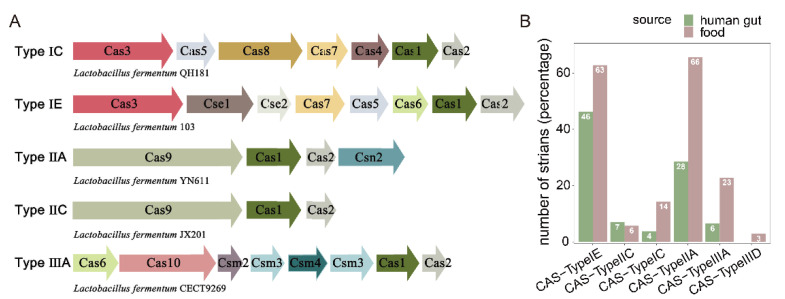
Clustered regularly interspaced short palindromic repeats (CRISPRs)/Cas system in *L. fermentum*. (**A**) Represent systems (Type IC, Type IE, Type IIA, Type IIC and Type IIIA) of Cas genes were listed; (**B**) distribution of CRISPR–Cas systems among strains of *L. fermentum* isolated from human gut and food.

CRISPRs and cas genes in the genomes of 224 *L. fermentum* strains were analyzed. The genomes of 210 *L. fermentum* strains contained at least one CRISPR, and the genomes of 159 *L. fermentum* strains included Cas genes (Table S3). Five CRISPR subgroups (Types IE, IIA, IIC, IIIA, and IC) were identified in 224 *L. fermentum* strains and class 1 Type IE was the most abundant subtype, followed by class 2 Type IIA (Table S3). Except for class 2 Type IIC, the abundant of CRISPR Types IE, IIA, IIIA, and IC were all higher in *L. fermentum* of food source compared with human gut source. Remarkably, *L. fermentum* strains of food source had significantly more CRISPR class 2 Type IIA and class 3 Type IIIA, which were almost 2.5 to 4 times more than that in *L. fermentum* derived from human gut (Figure 7). Phylogenetic analysis of *Cas1* and *Cas2* (differing by CRISPR subtype) showed that *Cas1* and *Cas2* genes variably distributed in *L. fermentum* that had nothing to do with their origin (Figure S1).

**Figure 8:**
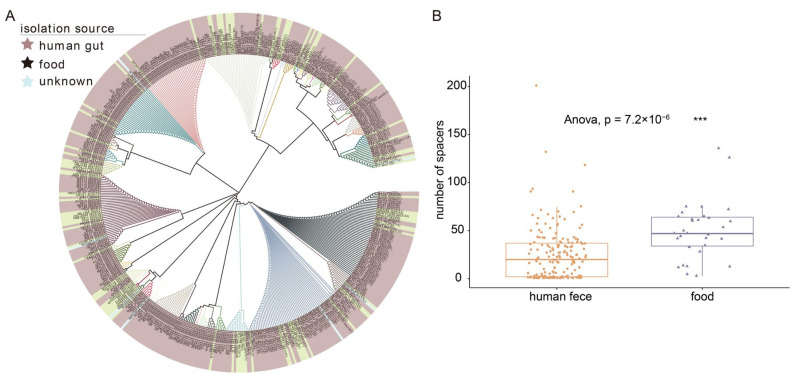
Distribution of the genome-targeting sequences in *L. fermentum*. (**A**) Phylogenetic analysis of spacer sequences in the genome of *L. fermentum* strains from human gut and food (Strains in the phylogenetic tree annotated with various leaf background colors correspond to different sources marked by stars in three different colors); (**B**) box plot showed the number of spacers in the genome of *L. fermentum* strains from two separate sources and * indicate a significant difference in the number of spacers between different group (*** *p* < 0.001).

Spacers are small fragments of foreign DNA incorporated into bacteria’s own CRISPR loci to avoid invasion by alien species. On the phylogenetic tree, spacers of *L. fermentum* clustered into nearly 50 phylogenetic groups (Figure 8A). Distinct spacers sequence of *L. fermentum* are color-coded in the branches of the phylogenetic tree and more abundant spacer sequences were contained in *L. fermentum* of human gut source. Some spacers were only owned by human source *L. fermentum* strains. The spacers gene abundance of 224 *L. fermentum* strains was analyzed using PERMANOVA and pairwise comparison and the number of spacers showed significant difference between two source groups. The number of spacers in food source *L. fermentum* was significantly higher than that in human gut source *L. fermentum* (Figure 8B).

### 3.6. Identification of Prophages in L. fermentum Strains

**Figure 9:**
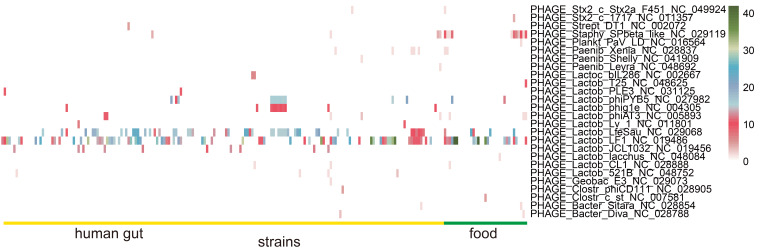
Identification of prophages in *L. fermentum*. Heatmap of the number of prophages in the genome of *L. fermentum* strains derived from human gut and food.

The number of prophages in *L. fermentum* strains predicted to be “intact” using PHASTER are shown in Figure 9. PHAGE_Lactob_LfeSau and PHAGE_Lactob_LF1 were the most abundant prophages in all *L. fermentum* strains and food source *L. fermentum* contained more abundant PHAGE_Staphy_SPbeta_like. Furthermore, less common prophages such as PHAGE_Paenib_Xenia, PHAGE_Lactob_phiPYB5, and PHAGE_Lactob_phig1e were distributed sporadically in *L. fermentum* of both sources. PHAGE_Lactob_JCL1032 and PHAGE_Lactob_521B were found only in “human gut source” *L. fermentum* strains.

## 4. Discussion

Research has shown that *Lactobacillus* species populate nutrient-rich habitats, such as fermented plant matter and in animals (both vertebrates and invertebrates, including humans) [6]. It is generally believed that microbes constantly evolve through gene variants and horizontal gene transfer between distinct microbes to face a range of selective pressures in a variety of ecological environments [38]. In this study, the average percentage identity between nucleotide sequences of 224 *L. fermentum* strains was more than 97%, while the Venn diagram showed that the maximum number of specific genes of *L. fermentum* reached 525 (Figure 1). Pan- and core-genome analyses also showed that as the number of *L. fermentum* strains increased, the number of pan genes increased and the number of core genes continued to shrink. Research also showed that lactobacilli in distinct habitats could evolve with their environment and generate unique genes [14]. We speculated that if the number of sources of *L. fermentum* increased, the curves of pan and core genes would become steeper. Good et al. showed that molecular evolution in *Escherichia coli* was dynamic, driven by the accumulation of mutations, and constantly created new genetic opportunities for adaptation of strains [39]. This may also explain the growing number of pan genes and numerous unique genes in the genome of *L. fermentum* strains.

Phylogenetic analysis of *L. fermentum* strains derived from human gut and food (such as yogurt, dairy, sourdough, kimchi, fermented plant material, and fermented meat) was conducted in this study, and 224 *L. fermentum* strains were mainly clustered into six clades (Figure 2). Most of the *L. fermentum* strains isolated from the human gut clustered in clade Ⅱ, while the rest were mainly found in clades Ⅰ, Ⅴ, and Ⅵ. Research over the past few decades has clarified that symbiotic microbes and their metabolites (SCFAs, endotoxins, peptidoglycans, and polysaccharide antigens) play a crucial role in defending against pathogen colonization, host physiology (immunoregulation), and metabolism, which is widely believed to be a result of coevolution [40]. Many factors, such as exposure to xenobiotics and host diet [41], may provide the host with unique selective pressures on its gut microbiota [42]. Filannino et al. showed that lactic acid bacteria in plant foods participated in a series of reactions (fatty acid metabolism, carbon metabolism, nitrogen metabolism, and phenolic metabolism) through specific bacterial enzymes (such as linoleate isomerase, fatty acid hydratases, mannitol dehydrogenase, reductase, and amine dehydrogenase), and the fermentation process relies on the rapid adaptation and metabolic capability of *Lactobacillus* with available nutrients [43]. Since the ecological environment of the human intestinal tract and food are distinct, the phylogenetic analysis of our study may illustrate the niche-specific adaptation of *L. fermentum* strains to different habitats. Batstone et al. explored whether the host could actively choose more cooperative microbial strains through a cross-inoculation experiment and the results showed that rhizobia rapidly adapted and gave preference to its original legume genotype, evolved to be more beneficial, and the process was not affected by host selection [44]. It is possible that the separation of *L. fermentum* strains from different sources is also the result of long-term coevolution between *L. fermentum* and its sources. We have to admit that, unfortunately, the number of food *L. fermentum* strains was lower than that of human feces strains. Based on the current studies, we think that the impact of the number of strains from different origin on our results was limited. Marko Verce et al. showed that 28 *L. fermentum* isolated from mammal tissues, milk, and plant material fermentations clustered into five clades and was independent of their sources [45]. Another study by Oh et al. indicated that evolution of *L. reuteri* lineages was adaptive for the different host species, although the sample numbers from different host were unequal (humans (n = 35), mice (n = 35), rats (n=26), pigs (n = 41), chickens (n = 26), and turkeys (n = 5)) [46]. Although phylogenetic analysis revealed that *L. fermentum* strains isolated from the human gut and food clustered separately, clade Ⅴ contained *L. fermentum* strains from both sources; additionally, these host-specific clusters (Ⅰ, Ⅱ, Ⅵ, human source; Ⅲ, Ⅳ, food source) contained some strains originating from other hosts. Pennisi reported that the widest range of microbes was found in soil and free-living samples, followed by plants, algae, and carnivores, and microbes could spread across host and habitats [47]. Pasolli et al. analyzed the relevance between 666 food source microbiomes and 154,723 human sample microbiomes and speculated that food was the main source of lactic acid bacteria in the human gut [48]. Food and the human gut could be regarded as open systems, and some *L. fermentum* might have been recently introduced and transient in the temporary environment. This may explain why some *L. fermentum* strains were promiscuous in host-specific clades.

The COG database, Initially created in 1997, has undergone a series of updates, currently including complete genomes of 122 archaea and 1187 bacteria, and is a popular tool for annotation of functional proteins [49]. An average of 2000 coding sequences was contained in 224 *L. fermentum* strains, and approximately 1400 COGs were annotated in the genome of these *L. fermentum* strains (Table S1). pCoA, PERMANOVA, and pairwise analysis of COGs in *L. fermentum* strains showed that significant differences existed between the food source and human source clades, and we believe that this could presumably reveal their relationship. Genes involved in mobilomes, prophages, and transposons (functional categories of X in COGs) were significantly higher in food source clades (Ⅲ and Ⅳ). Carr et al. showed that mobile genetic elements often move via horizontal gene transfer within a community [50] and studies have also shown that microbes in plants are more diverse than those in the human gut [47]. Wibowo et al. analyzed the microbial genomes from palaeofaeces samples and present-day human gut samples and indicated that mobile genetic elements in human gut microbiomes decreased over time [51].

Since the huge difference existed between human gut source and food source *L. fermentum* in both evolution and homologous genes, we then focused our attention on the specific differential genes belonging to unique environment. *L. fermentum* strains derived from human gut source contained significantly more dominant COG categories and these main COG classes were related to various functions including energy production and conversion (C), amino acid transport and metabolism (E), carbohydrate transport and metabolism (G), transcription (K), replication, recombination, and repair (L), and general function prediction only (R) (Table S2). Hao Luo analyzed the Ka/Ks ratio of genes in functional categories of COGs and found that genes in functional categories of G, H, I, J, K, and L were more evolutionarily conserved and were more essential in coping with strong selective pressure [52]. Perhaps the genome-scale differences in *L. fermentum* were due to the individual evolution in host gut niche, reflecting the specific host physiology or dietary habits [53]. Food source *L. fermentum* had more genes encoding transposase. We speculated that the microbiota was more complex in the food than those in the human gut, and *L. fermentum* strains from food sources were more easily exposed to the mobile genetic elements.

Unique metabolic capacity is highly associated with the adaptation of microorganisms to their specific niche [54]. CAZy analysis showed that a total of 38 carbohydrate active enzyme families existed in 224 *L. fermentum* strains and the distribution of these enzymes contained in *L. fermentum* was previously unknown. Research shows that *L. crispatus* [55] and *L. reuteri* [46] had 59 and 54 kind of carbohydrate active enzyme families (GHs, GTs, PLs, CEs, AAs, and CBMs). Compared to these *Lactobacillus* species, *L. fermentum* had a simpler set of carbohydrate enzyme families and GT2, GT4, GH73, and CBM50 were major families. A previous report showed that dominant carbohydrate enzyme families in *L. plantarum* were CBM50, GH1, GH2, and GT4 [56], which may indicate that the distribution of carbohydrate varied between different *Lactobacillus* species. The abundance of these families in *L. fermentum* with different sources were also discrepant and the number of genes encoding enzymes for degrading carbohydrates in food source *L. fermentum* was statistically richer than that of human gut source, which may imply that food source *L. fermentum* had stronger metabolic function. Carbohydrate-active enzyme families GT2, GH 43_11, and GH 68 were more abundant in food source *L. fermentum* strains and human gut *L. fermentum* strains had a higher number of enzymes in families GH3, GH13_20, and GH13_29, while there was little variation on the kinds of carbohydrate active enzyme families in *L. fermentum* of two groups. Hehemann et al. showed that seaweeds are an important daily diet item in Japan, and that genes coding for porphyranases and agarases in *Zobellia galactanivorans* (a member of the marine *Bacteroidetes* in seaweeds) were transferred to Japanese gut bacteria [57]. Maria et al. also showed that host’s diet is a key evolutionary force shaping gut microbiota and influences the evolution trend of gut symbionts [58]. The genomic diversity of human feces source *L. fermentum* may also be associated with differences in the host’s diet. This suggests that the enzymes involved in the metabolism of carbohydrates in the microbiota of the human gut are inextricably linked to that of food. Then, the metabolic pathways of some common sugars and genes encoding AraD (arabinose), bglX (cellobiose), XylA (xylose) and deoB (ribose) were separately more abundant in certain sources. Martino et al. showed that the diet of the host could shape the evolutionary direction of its symbiotic bacteria [58]. Whether the difference of nutritional environment leads to the result needs to be further studied.

Genes conferring resistance to daptomycin (*cls*, *pgsA*), isoniazid and triclosan (*fabI*), fosfomycin (*GlpT* and *murA*), fluoroquinolones (*gyrA* and *gyrB*), mupirocin (*mupB* and *mupA*), amoxicillin (*PBP2x*), kirromycin (*EF-Tu*), rifampicin (*rpoB*), and fusidic acid (*fusA*) were found in almost all *L. fermentum* strains in this study. A previous report showed that mobile genetic elements were highly related to the spread of antimicrobial-resistance genes [50]. We have mentioned that genes involved in mobilome, prophages, and transposons were dominant in *L. fermentum* strains isolated from food and this may be linked to antibiotic resistance genes. The common use of pesticide adjuvants in agricultural activities [59] may be one of the reasons for the larger number of antibiotic resistance genes in food source *L. fermentum*. The number of genes related to resistance to tetracycline antibiotic (*otr(A)*, *tetA(46),* and *poxtA*), lincosamide antibiotic (*lmrB*), fluoroquinolone, rifamycin, and macrolide antibiotic (*efrB*) was relatively high in food source *L. fermentum* strains and the genes (*pmrA*, *bcrA, arlR*, *vanRF,* and *mdtG*), which confer resistance to fluoroquinolone antibiotic, peptide antibiotic, fluoroquinolone antibiotic, glycopeptide antibiotic, and phosphonic acid antibiotic, were prominently present in human gut source *L. fermentum*. Sommer et al. showed that the evolution of antibiotic resistance was driven by mutations and horizontal gene transfer between different bacteria and horizontal gene transfer was the major factor [60]. Debroas et al. indicated that viruses connected to putative pathogens (*Enterobacterales* and *Vibrionaceae*) were the major medium to transfer antibiotic resistance genes [61]. Therefore, the difference in antibiotic resistance genes between distinct clades may be due to the use of antibiotics [62], the presence of viruses, and bacterial diversity in the environment.

CRISPRs and CRISPR-associated Cas genes could prevent the microbiota from bacteriophage and foreign DNA infections and were an important defense strategy for bacteria [63]. Of the 224 *L. fermentum* strains, 210 (over 90%) contained at least one CRISPR. Sun et al. analyzed the presence of CRISPR loci in 213 *Lactobacillus* strains, and their results showed that 62.9% of the strains contained the CRISPR loci [64]. Subtype IE CRISPR-Cas systems were most common in *L. fermentum* and Cas (Cas1 and Cas2) protein sequence comparison showed that the Cas genes were randomly distributed in the phylogenetic tree independent of their origin (Figure S1). Studies have also shown that Cas1 and Cas2 are the most conserved protein components in the CRISPR-Cas systems [63,65]. A previous report indicated that of the six types of CRISPR-Cas systems (Types I, II, III, IV, V, and VI), Type I was most widely distributed in bacilli [66]. Our results showed that the number of spacers differed between certain origins and food source *L. fermentum* had significantly more spacers. Cantabrana et al. showed that CRISPR spacers could be exploited to provide insights into host–phage interactions within a niche [67]. The proportion of strains containing phage was the highest in food source *L. fermentum*, which may be linked to the large number of spacers within (Figure 9). However, since the number of food *L. fermentum* strains was lower than that of human feces strains, we could not rule out the possibility that this was the reason for some spacers only found in human source *L. fermentum* strains. In addition, prophage Staphy_SPbeta_like, which was of Staphylococcus origin, was dominant in food source *L. fermentum*, which may indicate the existence of Staphylococcus in certain niches. Prophage Lactob_LF1 [68] and Lactob_LfeSau [69], previously reported to be present in the genome of *L. fermentum*, were abundant in all of these *L. fermentum* strains.

## 5. Conclusions

In summary, our results showed that 224 *L. fermentum* strains contained 20,505 pan gene families and 615 core gene families. These strains mainly clustered into six clades (I, II, III, IV, V, and VI) in the phylogenetic tree and there was a tendency of clustering with origin (human gut and food). Homologous genes related to mobilomes, prophages, and transposons were dominant genes in *L. fermentum* strains derived from food and human gut source *L. fermentum* had more genes with various functions. Furthermore, genes belonging to carbohydrate enzyme family GT2, GH 43_11, and GH 68 were more abundant in food source *L. fermentum* strains and family GH3, GH13_20, and GH13_29 were commonly seen in human gut *L. fermentum* strains. The number of genes encoding *otr(A)*, *tetA(46), poxtA*, *lmrB,* and *efrB* was relatively high in food source *L. fermentum* strains and the genes referring to pmrA, bcrA, arlR, vanRF, and mdtG were prominently present in human gut source *L. fermentum*. The number of CRISPR spacers in “food source” *L. fermentum* was significantly higher than that in human gut source *L. fermentum* and food source *L. fermentum* contained more abundant prophage of PHAGE_Staphy_SPbeta_like, which could provide strong evidence for the adaptive capacity and evolution process of *L. fermentum* strains in different niches. In general, the genomic and metabolic analysis of food source and human feces source *L. fermentum* may provide valuable information for the industrial and therapeutic applications of *L. fermentum* in the future.

## Supplementary Materials

The following supporting information can be downloaded at: https://www.mdpi.com/article/10.3390/foods11193135/s1, Table S1: Genomic information and detailed source of 224 *L. fermentum* strains in the study. Table S2: LDA score of dominant COG categories in the genome of *L. fermentum* strains derived from human gut and food. Table S3: Distribution of CRISPR-Cas systems in the genome *L. fermentum* strains. Figure S1: Phylogenetic analysis of Cas1 and Cas2 genes in the genome of *L. fermentum* from human gut and food.
