# Supplementary material for: Environment-Related Genes Analysis of Limosilactobacillus fermentum Isolated from Food and Human Gut: Genetic Diversity and Adaption Evolution
Source: Foods. 2022 Oct 8;11(19):3135. doi: 10.3390/foods11193135 (PMC9564382; doi:10.3390/foods11193135)

Supplementary Materials

Table S1: Genomic information and detailed source of 224 *L. fermentum* strains in the study.

strain	accession	isolation source	age	sex	total length (Mb)	GC%	CDS
AH451	SRR12560031	human feces	6	female	1.92004	52.162	1901
AH471	SRR12559885	human feces	86	male	1.983962	51.9	1916
BJ74	SRR12559874	human feces	62	male	1.923224	52.173	1904
BJ204	SRR12559863	human feces	61	male	2.01921	51.927	1961
BJ211	SRR12559486	human feces	52	female	1.929755	52.029	1882
BJ241	SRR12559475	human feces	18	male	1.972608	52.084	1905
BJ314	SRR12559463	human feces	54	male	2.029152	51.875	1963
BJ361	SRR12559753	human feces	75	female	1930506	52.286	1919
BJ381	SRR12559644	human feces	50	male	1.942296	51.58	1933
BJ431	SRR12559643	human feces	53	male	2.002277	51.94	2011
BJ581	SRR12559642	human feces	55	male	2.025549	51.78	2024
BJ613	SRR12559641	human feces	85	male	2.155527	51.5	2216
BJ631	SRR12559640	human feces	81	male	1.975164	52.09	1982
CQ272	SRR12559637	human feces	73	female	1.973607	52.08	1977
CQ61	SRR12559742	human feces	77	male	2.011778	51.93	1964
CQ71	SRR12559731	human feces	67	male	2.034302	51.987	1983
CQ91	SRR12559720	human feces	53	female	1.91	52.3	1986
CQ151	SRR12559638	human feces	48	male	2.030093	51.89	2045
CQ309	SRR12559709	human feces	84	female	1.946381	52.298	1877
CQ368	SRR12559698	human feces	13	female	1.94974	52.215	1866
FJ112	SRR12559636	human feces	1	male	2.015384	51.72	2002
FJ12	SRR12559984	human feces	1	male	1.969459	52.125	1906
FJ16	SRR12559973	human feces	1	male	2.00127	51.972	1973

Foods

FJ181	SRR12560053	human feces	1	male	2.110157	51.571	2008
FJ191	SRR12560042	human feces	1	female	1.964027	52.011	2003
GD111	SRR12559962	human feces	77	male	1.983958	52.04	1980
GD121	SRR12559821	human feces	46	male	1.985253	52.05	1972
GD131	SRR12559809	human feces	12	male	1.979319	52.07	1969
GD181	SRR12559798	human feces	42	male	1.962846	52.11	1959
GD223	SRR12560116	human feces	10	male	2.077208	51.63	2097
GD331	SRR12560105	human feces	86	male	1.922951	52.14	1926
GD351	SRR12559847	human feces	77	female	2.053515	51.82	2088
GD363	SRR12559846	human feces	78	female	2.082809	51.57	2121
GD611	SRR12559845	human feces	74	male	2.035065	51.83	2014
GD661	SRR12559844	human feces	9	male	2.087264	51.56	2078
GD673	SRR12559843	human feces	64	female	2.036044	51.79	2022
GD712	SRR12559647	human feces	72	female	2.071553	51.81	2045
GD723	SRR12559646	human feces	70	female	1.947764	52.02	1942
GD741	SRR12560025	human feces	10	male	1.949121	52.02	1941
GD752	SRR12560024	human feces	68	male	1.930005	52.24	1903
GD761	SRR12559645	human feces	65	female	2.010427	52.17	1994
GS504	SRR12559949	human feces	13	male	1.958086	52.216	1859
GX51	SRR12559938	human feces	83	female	1.953285	51.89	1960
GX61	SRR12559927	human feces	81	female	2.035179	51.68	2069
HM107	SRR12559842	human feces	87	female	2.035179	51.68	2069
HM301	SRR12559494	human feces	71	female	1.98874	51.924	1939
HM311	SRR12559585	human feces	65	female	1.925204	52.098	1891
HM321	SRR12559574	human feces	86	female	1.97109	52.056	1916
HM661	SRR12559517	human feces	50	female	1.931465	52.282	1863
HN112	SRR12559658	human feces	92	male	2.144258	51.33	2175

Foods

HN114	SRR12559657	human feces	92	male	2.14437	51.32	2173
HN115	SRR12559656	human feces	92	male	2.142472	51.34	2168
HN116	SRR12559655	human feces	92	male	2.144719	51.32	2173
HN117	SRR12559654	human feces	92	male	2.133678	51.38	2165
HN118	SRR12559653	human feces	92	male	2.020379	51.96	2034
HN119	SRR12559652	human feces	92	male	2.119819	51.44	2130
HN1110	SRR12559651	human feces	92	male	2.131763	51.39	2163
HN286	SRR12559837	human feces	48	male	2.01399	52.031	1914
HN441	SRR12559649	human feces	1	female	1.977274	52.16	2002
HN191	SRR12559841	human feces	77	female	2.020119	51.77	2032
HN239	SRR12559840	human feces	72	female	1.925493	52.27	1918
HN261	SRR12559839	human feces	66	female	1.981315	51.99	1979
HN401	SRR12559836	human feces	3	female	1.975071	51.93	1965
JX111	SRR12559544	human feces	61	female	2.049902	51.863	2014
JX121	SRR12559543	human feces	72	male	1.973114	52.182	1878
JX192	SRR12559542	human feces	71	female	2.049329	52.025	1933
JX201	SRR12559541	human feces	73	female	2.021685	51.915	1959
JX61	SRR12559545	human feces	12	female	1.963315	52.155	1906
NM232	SRR12559540	human feces	51	female	1.973519	52.107	1887
NM241	SRR12559538	human feces	51	male	1.867068	52.487	1828
NM271	SRR12559537	human feces	41	female	2.104537	51.587	1922
NT21	SRR12559832	human feces	90	female	2.012898	52.004	1885
NT272	SRR12559831	human feces	78	female	1.992488	52.261	1891
NT31	SRR12559830	human feces	72	male	2.030522	51.77	2041
NT41	SRR12559829	human feces	73	female	1.929138	51.7	1919
NT652	SRR12559826	human feces	85	male	2.149885	51.616	1964
NT153	SRR12559835	human feces	95	female	1.958412	52.03	1955

Foods

NT165	SRR12559834	human feces	82	female	2.023924	51.71	2046
NT171	SRR12559833	human feces	/	/	1.982732	51.97	1991
NT575	SRR12559828	human feces	74	male	2.123841	51.37	2166
NT753	SRR12559563	human feces	91	female	2.107499	51.62	2103
NX642	SRR12559634	human feces	58	male	2.050731	51.76	2096
NX657	SRR12559633	human feces	65	female	2.257901	51.14	2315
NX681	SRR12559632	human feces	17	male	2.02146	51.92	2046
QH161	SRR12559536	human feces	10	female	1.933043	52.17	1901
QH181	SRR12559535	human feces	46	male	1.936134	52.135	1866
QH221	SRR12559534	human feces	38	female	1.959191	52.156	1872
QH495	SRR12559533	human feces	33	male	1.947097	52.214	1861
QH804	SRR12559532	human feces	/	male	2.108428	51.493	1923
QH837	SRR12559531	human feces	37	male	2.159499	51.436	2022
SC381	SRR12559530	human feces	/	male	1.957259	52.314	2059
SH101	SRR12559990	human feces	82	female	2.064394	51.931	2005
SD131	SRR12559631	human feces	1	female	2.045336	51.73	2066
SH171	SRR12559693	human feces	86	female	2.056894	51.845	1951
SH251	SRR12559857	human feces	86	female	1.930566	52.22	1922
SH272	SRR12559856	human feces	85	female	1.987704	51.94	1964
SH281	SRR12559855	human feces	85	female	1.96091	52.04	1947
SH371	SRR12559854	human feces	83	female	2.023529	51.8	2020
SH43	SRR12560125	human feces	86	female	1.947462	52.342	1866
SH54	SRR12560124	human feces	85	female	2.026251	51.816	1954
SH65	SRR12560123	human feces	87	male	2.035882	51.787	1951
SH95	SRR12560122	human feces	90	male	2.087559	51.657	1996
SL1321	SRR12559630	human feces	72	female	1.885286	52.07	1865
SL185	SRR12559629	human feces	52	female	2.127545	51.32	2146

Foods

SL211	SRR12559526	human feces	78	male	1.975867	52.107	1910
SL2213	SRR12559525	human feces	55	male	1.953168	52.214	1868
SL241	SRR12559524	human feces	75	female	1.980236	51.933	1874
SL271	SRR12559789	human feces	68	female	1.892259	52.436	1828
SL292	SRR12560126	human feces	76	female	1.996618	52.056	1920
SL312	SRR12559627	human feces	68	female	2.059861	51.71	2090
SL521	SRR12559626	human feces	60	male	1.883842	52.34	1852
SP241	SRR12559529	human feces	100	female	1.890399	52.275	1863
SP331	SRR12559527	human feces	54	male	1.940669	52.329	1839
WX111	SRR12559691	human feces	78	female	2.022235	51.88	2026
WX112	SRR12559690	human feces	78	female	2.021786	51.88	2017
WX113	SRR12559689	human feces	78	female	2.020754	51.87	2007
WX114	SRR12559688	human feces	78	female	2.020046	51.87	2018
WX115	SRR12559687	human feces	78	female	2.01993	51.87	1999
WX11	SRR12559785	human feces	80	/	1.925893	52.267	1874
WX121	SRR12559553	human feces	84	female	1.971969	51.987	1903
WX141	SRR12559552	human feces	78	male	1.961175	52.208	1854
WX161	SRR12559551	human feces	89	male	2.132632	51.61	2037
WX183	SRR12559549	human feces	81	male	1.985916	52.096	1896
WX213	SRR12559548	human feces	90	female	1.922467	52.288	1828
WX252	SRR12559547	human feces	84	female	2.020009	51.891	1951
WX61	SRR12559774	human feces	81	female	1.9747	52.131	1877
WX91	SRR12559763	human feces	85	female	2.005265	51.936	1941
XC263	SRR12559684	human feces	56	female	1.964818	52.232	1900
XC32	SRR12559686	human feces	2	female	2.086295	51.69	2100
XC416	SRR12559682	human feces	26	female	1.942235	52.261	1837
XC61	SRR12559685	human feces	5	male	2.116212	51.45	2131

Foods

XS212	SRR12559681	human feces	53	female	1.937229	52.322	1898
XS71	SRR12559680	human feces	11	male	2.02987	51.59	2061
XW241	SRR12559679	human feces	45	female	2.148222	51.724	2012
XW331	SRR12559678	human feces	36	female	1.959917	52.17	2010
XW341	SRR12559677	human feces	57	female	1.965164	52.17	2022
XW391	SRR12559676	human feces	7	female	2.010074	51.63	1998
XW411	SRR12559675	human feces	8	male	2.007773	51.92	1989
XW463	SRR12559674	human feces	48	female	2.026653	51.908	2035
YC74	SRR12559546	human feces	/	female	1.998299	52.046	2013
YN361	SRR12560023	human feces	/	female	2.119972	51.23	2131
YN497	SRR12560022	human feces	/	/	2.036369	51.5	2091
YN54	SRR12559673	human feces	/	/	1.958265	52.304	1892
YN64	SRR12559671	human feces	49	female	2.103536	51.582	2014
YN96	SRR12559670	human feces	57	male	1930423	52.241	1825
YN611	SRR12559669	human feces	11	female	2.107499	51.62	2103
YZ156	SRR12560021	human feces	60	male	1.863906	52.3	1872
YZ1610	SRR12560020	human feces	59	male	1.969128	52.2	1995
YZ96	SRR12559635	human feces	63	male	1.970882	51.9	1947
ZH1010	SRR12559853	human feces	/	/	1.986303	51.7	1953
ZH241	SRR12559852	human feces	/	male	1.968883	51.99	1970
ZJ122	SRR12560019	human feces	7	female	2.187189	51.24	2238
ZT25	SRR12559668	human feces	65	female	1.95084	52.193	1868
ZT139	SRR12559667	human feces	76	female	1.938162	52.085	1877
ZT205	SRR12559666	human feces	83	male	1.930131	52.461	1861
ZT226	SRR12559665	human feces	/	female	1.929055	52.413	1938
ZT238	SRR12559664	human feces	/	male	1.948108	52.333	1957
ZT243	SRR12559663	human feces	62	female	2.016053	51.965	1990

Foods

ZT251	SRR12559662	human feces	80	male	2.013958	51.904	1936
ZT591	SRR12559851	human feces	/	male	2.084569	51.67	2078
ZT607	SRR12559850	human feces	78	female	2.082168	51.57	2101
ZT618	SRR12559848	human feces	75	male	1.989632	51.97	1992
ZT671	SRR12559660	human feces	83	male	2.010001	51.919	1945
ZT681	SRR12559659	human feces	/	male	2.020224	51.869	1968
LMT2_75	online	kimchi			2.33	50.48	2,108
2760	online	dairy			2.27	51.4	2,004
FTDC_8312	online	fecal sample			2.24	51	2,029
USM_8633	online	fermented meat sausage			2.24	51	2,028
SRCM103285	online	food			2.15	51.3	1,979
SRCM103290	online	food			2.12	51.4	1,863
HFD1	online	Homo sapiens fecal sample			2.1	51.8	1,927
IMDO130101	online	sourdough			2.09	51.5	1,835
LDTM7301	online	Makgeollli			2.05	51.7	1,837
CBA7106	online	adult feces			2.04	51.7	1,859
MTCC25067	online	fermented milk			2.01	51.18	1,684
NCC2970	online	/			1.95	52.2	1,758
YL_11	online	fermented milk			1.91	51.9	1,656
B128	online	fermented beets			1.91	52.3	1,736
MTCC5898	online	Homo sapiens Infant fecal			2.1	52.1	1,583

Foods

		sample			
VRI_003	online	Commercial probiotic culture lyophilized	1.95	52	1817
47_7	online	Homo sapiens fecal sample	2.1	52.2	1,644
LFQI6	online	Homo sapiens fecal sample	2.1	52.1	1,623
39	online	human feces	1.83	51.6	1,704
CRL1446	online	Goat milk cheese	2.15	51.4	1,976
D12	online	Fresh smoked cheese	2.02	52	1,868
222	online	Cocoa bean fermentation	1.95	52.1	1,768
AF15_40LB	online	human feces	1.97	52	1,813
MGYG_HGUT_00166	online	human gut	1.97	52	1,814
FUA3588	online	Mahewu	2.02	51.7	1,840
S6	online	Sour wort, Tchapalo (Sorghum african beer) processing	1.91	52.3	1,718
S13	online	Sour wort, Tchapalo (Sorghum	1.92	52.3	1,718

Foods

		african beer) processing			
AF11_4_H	online	human fece	1.94	52.2	1,380
103	online	Homo sapiens cecum	2.05	51.8	1,900
L13	online	human fece	1.95	52.6	1,821
279	online	Homo sapiens fece	1.98	52	1,828
DS19_7	online	dietary supplement products	2.02	51.6	1,807
311	online	Homo sapiens feces	2.04	51.8	1,860
317	online	fermented milk	1.92	51.5	1,674
CECT9269	online	tocosh, Peruvian traditional fermented potatoes	2.08	51.7	1,907
S30	online	human feces	2.16	51.2	2,008
L18	online	human feces	2.11	52	1,966
UCO_979C	online	Homo sapiens feces	2.01	51.9	1,517
DS13_7	online	dietary supplement products	2	51.8	1,770
AF16_22LB	online	human feces	2	51.9	1,827

Foods

LFU21	online	Homo sapiens feces of an astronaut	1.97	51.7	1,796
NBRC3959	online	/	1.93	52.1	1,831
BFE6620	online	Gari	1.98	52.1	1,806
KMB_612	online	bryndza cheese	1.92	52.2	1,764
FAM19471	online	cheese	2.04	51.5	1,921
RI_508	online	cacao been fermentation	1.92	52.2	1,767
KMB_613	online	bryndza cheese	2.01	52.1	1,875
FUA3589	online	Mahewu	2.08	51.5	1,843
LF2	online	dairy (cheese)	2.05	51.7	1,875
NCDC400	online	curd	1.9	51.6	1,660
DR9	online	Cow Fresh milk	2.36	50.44	1,678
HFB3	online	Homo sapiens fecal sample	2.04	51.8	1,366
CIMMAG1415	online	human digestive tract	1.76	49.7	1,750
3872	online	Homo sapiens milk from female	2.33	50.56	2111
ATCC14931	online	fermented beets	1.87	52.6	1,676
CECT5716	online	Human milk	2.1	51.5	1631
F_6	online	/	2.06	51.7	1,848
IFO3956	online	Fermented plant material	2.1	51.5	1916
LF1	online	Homo sapiens	1.82	52.5	1,677

Foods

		male human gut			
		feces			
MTCC8711	online	yogurt	2.57	49.65	2,264

Foods

Table S2: LDA (linear discriminant analysis) score of dominant COG categories in the genome of *L. fermentum* strains derived from human gut and food.

Food source	COG category	LDA score	Human gut source	COG category	LDA score
COG2826	X	3.672093952	COG1309	K	2.856816
COG3328	X	3.670942152	COG1028	I	2.770695
COG2801	X	3.547536632	COG0583	C	2.712504
COG0675	X	3.466125672	COG0531	E	2.685298
COG1943	X	3.376002135	COG0716	C	2.618832
COG2963	X	3.258079384	COG1063	E R	2.513247
COG3464	X	3.129916415	COG2151	O	2.492278
COG3436	X	2.664887429	COG0076	E	2.465703
COG3293	X	2.491585793	COG1136	M	2.450191
COG2217	P	2.45177289	COG3104	E	2.441617
COG1518	V	2.430339874	COG1062	C	2.40334
COG0789	K	2.421040582	COG0697	G E R	2.401072
COG0463	M	2.38417996	COG1073	T	2.399626
COG2814	G	2.292681744	COG0366	G	2.38801
COG0657	I	2.261533651	COG0745	K T	2.354477
COG2055	C	2.258852922	COG0577	V	2.345387
COG3051	C	2.255829674	COG0075	E F	2.333589
COG1767	H	2.249459398	COG0642	T	2.29508
COG3512	S	2.248213512	COG0436	E	2.277372
COG1397	O	2.183101012	COG1387	E R	2.267052
COG3513	V	2.159519355	COG0141	E	2.251421
COG5542	G	2.149190239	COG0118	E	2.246166
COG4152	R	2.128542946	COG0106	E	2.24565
COG0446	I	2.11520244	COG0390	P	2.244486
COG3378	X	2.076088223	COG1484	L	2.241856
COG1457	F	2.044253358	COG0040	E	2.239593
COG2190	G	2.033332489	COG3705	E	2.2371
COG2132	D M P	2.017478116	COG1760	E	2.233829
COG0074	C	2.016924426	COG0494	V	2.22388
COG5039	M G	2.013502484	COG0131	E	2.222522
COG0003	P	2.01101159	COG0473	C E	2.219941
human feces			COG3971	Q	2.213778
			COG0549	E	2.212223
			COG2188	K	2.209438
			COG0385	R	2.204749

Foods

			COG1139	C	2.203255
			COG0069	E	2.195191
			COG0798	P	2.191073
			COG3093	V	2.181763
			COG0065	E	2.181393
			COG0129	G E	2.174447
			COG0066	E	2.170776
			COG0154	J	2.166691
			COG4908	R	2.165513
			COG0059	E H	2.158818
			COG0039	C	2.158164
			COG1940	K G	2.149748
			COG1171	E	2.147106
			COG1959	K	2.136676
			COG1119	P	2.134307
			COG2350	Q R	2.132306
			COG0493	E R	2.131612
			COG1668	C P	2.127838
			COG3048	E	2.115627
			COG1808	S	2.086555
			COG1783	X	2.080448
			COG0394	T	2.079559
			COG0679	R	2.077975
			COG0340	H	2.074603
			COG0505	E F	2.073012
			COG0457	R	2.072303
			COG4690	E	2.07029
			COG1556	C	2.069592
			COG1263	G	2.058164
			COG1893	H	2.05467
			COG0507	L	2.053786
			COG0235	G E	2.042423
			COG0389	L	2.026598
			COG0846	O	2.023163
			COG1038	C	2.016359
			COG0550	L	2.016075
			COG0247	C	2.012152
			COG0790	R	2.00463
			COG2723	G	2.00168

Foods

Table S3: Distribution of CRISPR-Cas systems in the genome of *L. fermentum* strains (Of 224 *L. fermentum* strains, 210 strains contain at least one CRISPR and 159 strains containing cas genes are listed below).

strains	CAS Type	strains	CAS Type	strains	CAS Type
103	CAS-TypeIE	AF11_4_H	CAS-TypeIE	LMT2_75	CAS-TypeIIA
BJ431	CAS-TypeIIC	ZT671	CAS-TypeIE	SL185	CAS-TypeIIA
HN261	CAS-TypeIIA	JX111	CAS-TypeIE	SL185	CAS-TypeIE
ZT618	CAS-TypeIIA	JX192	CAS-TypeIE	QH495	CAS-TypeIE
ZT618	CAS-TypeIIC	YZ1610	CAS-TypeIE	NM241	CAS-TypeIIA
BJ74	CAS-TypeIIA	GD223	CAS-TypeIE	NM232	CAS-TypeIIA
CBA7106	CAS-TypeIE	QH837	CAS-TypeIE	NM232	CAS-TypeIE
SRCM103285	CAS-TypeIIA	XC32	CAS-TypeIE	NM232	CAS-TypeIIC
LDTM7301	CAS-TypeIIA	GD661	CAS-TypeIE	XS71	CAS-TypeIIA
LDTM7301	CAS-TypeIE	GD661	CAS-TypeIIA	XS71	CAS-TypeIE
SL211	CAS-TypeIE	GD661	CAS-TypeIIC	XS71	CAS-TypeIIC
YN611	CAS-TypeIIA	GD673	CAS-TypeIE	XS71	CAS-TypeIE
QH181	CAS-TypeIE	GD673	CAS-TypeIIA	ZH1010	CAS-TypeIIC
QH181	CAS-TypeIC	GD673	CAS-TypeIIC	ZH1010	CAS-TypeIE
AF15_40LB	CAS-TypeIIIA	SH281	CAS-TypeIC	ZH1010	CAS-TypeIIIA
AF15_40LB	CAS-TypeIIA	JX201	CAS-TypeIE	ZH1010	CAS-TypeIIA
MGYG_HGUT_00166	CAS-TypeIIA	JX201	CAS-TypeIIC	317	CAS-TypeIIIA
MGYG_HGUT_00166	CAS-TypeIIIA	JX201	CAS-TypeIIA	NCDC400	CAS-TypeIIA
HM661	CAS-TypeIIA	YN64	CAS-TypeIE	NCDC400	CAS-TypeIE
HM661	CAS-TypeIE	ZT205	CAS-TypeIE	MTCC25067	CAS-TypeIC
AH451	CAS-TypeIE	SH54	CAS-TypeIE	MTCC8711	CAS-TypeIE
NT165	CAS-TypeIIA	WX91	CAS-TypeIE	MTCC8711	CAS-TypeIC
NT165	CAS-TypeIE	GD712	CAS-TypeIIA	MTCC8711	CAS-TypeIE
YN497	CAS-TypeIE	CQ91	CAS-TypeIE	YL_11	CAS-TypeIIA
SH251	CAS-TypeIIIA	CQ91	CAS-TypeIIC	YL_11	CAS-TypeIIID
F_6	CAS-TypeIE	CQ91	CAS-TypeIIA	YL_11	CAS-TypeIE
GD611	CAS-TypeIE	D12	CAS-TypeIIA	YZ96	CAS-TypeIIC
GD752	CAS-TypeIE	SL241	CAS-TypeIE	YZ96	CAS-TypeIIA
GD762	CAS-TypeIE	SL241	CAS-TypeIIA	BFE6620	CAS-TypeIE
NCC2970	CAS-TypeIIA	SL241	CAS-TypeIIIA	HN239	CAS-TypeIIA
GS504	CAS-TypeIIC	AF16_22LB	CAS-TypeIIIA	222	CAS-TypeIIC
GS504	CAS-TypeIIA	AF16_22LB	CAS-TypeIIA	YN54	CAS-TypeIIA
QH221	CAS-TypeIIA	S30	CAS-TypeIIA	SL2213	CAS-TypeIIA
HN401	CAS-TypeIIA	L18	CAS-TypeIIA	GX51	CAS-TypeIE
HN401	CAS-TypeIE	HN286	CAS-TypeIIA	BJ211	CAS-TypeIIA
SL312	CAS-TypeIE	HN286	CAS-TypeIIIA	DS13_7	CAS-TypeIIC
IFO3956	CAS-TypeIE	SH65	CAS-TypeIE	DS13_7	CAS-TypeIE

Foods

IFO3956	CAS-TypeIC	SH65	CAS-TypeIIIA	DS13_7	CAS-TypeIIA
NBRC3959	CAS-TypeIE	SH95	CAS-TypeIE	DS19_7	CAS-TypeIIA
NBRC3959	CAS-TypeIC	SH95	CAS-TypeIIIA	DS19_7	CAS-TypeIE
NT753	CAS-TypeIE	ZJ122	CAS-TypeIIA	SRCM103290	CAS-TypeIE
NX657	CAS-TypeIE	ZJ122	CAS-TypeIIIA	SRCM103290	CAS-TypeIIA
QH804	CAS-TypeIE	2760	CAS-TypeIIA	IMDO130101	CAS-TypeIIA
XW241	CAS-TypeIE	2760	CAS-TypeIIIA	IMDO130101	CAS-TypeIE
WX161	CAS-TypeIE	2760	CAS-TypeIE	FUA3588	CAS-TypeIE
XC416	CAS-TypeIE	3872	CAS-TypeIIA	FUA3588	CAS-TypeIIIA
XC61	CAS-TypeIIA	3872	CAS-TypeIC	FUA3589	CAS-TypeIE
XC61	CAS-TypeIC	KMB_612	CAS-TypeIIA	FUA3589	CAS-TypeIC
XW331	CAS-TypeIIIA	FTDC_8312	CAS-TypeIIA	311	CAS-TypeIC
XW341	CAS-TypeIIIA	FTDC_8312	CAS-TypeIE	311	CAS-TypeIIA
NM271	CAS-TypeIIA	USM_8633	CAS-TypeIIA	GD331	CAS-TypeIIA
WX213	CAS-TypeIE	USM_8633	CAS-TypeIE	GD723	CAS-TypeIIA
WX61	CAS-TypeIE	VRI_003	CAS-TypeIIA	GD741	CAS-TypeIIA
AH471	CAS-TypeIE	VRI_003	CAS-TypeIE	SL521	CAS-TypeIE
BJ631	CAS-TypeIE	FAM19471	CAS-TypeIIA	SP241	CAS-TypeIE
HM107	CAS-TypeIE	FAM19471	CAS-TypeIE	ZT139	CAS-TypeIE
BJ581	CAS-TypeIE	YN361	CAS-TypeIE	HM311	CAS-TypeIIA
SH272	CAS-TypeIE	YN361	CAS-TypeIIC	HM311	CAS-TypeIE
CQ61	CAS-TypeIE	YN361	CAS-TypeIIA	HM321	CAS-TypeIE
NT41	CAS-TypeIE	YN361	CAS-TypeIC	WX11	CAS-TypeIE
39	CAS-TypeIE	CECT9269	CAS-TypeIE	WX114	CAS-TypeIIA
NT575	CAS-TypeIE	CECT9269	CAS-TypeIIA	WX114	CAS-TypeIE
BJ204	CAS-TypeIE	CECT9269	CAS-TypeIIIA	WX113	CAS-TypeIIA
NX642	CAS-TypeIE	CRL1446	CAS-TypeIIA	WX113	CAS-TypeIE
XW391	CAS-TypeIE	CRL1446	CAS-TypeIE	WX112	CAS-TypeIIA
XW391	CAS-TypeIE	CRL1446	CAS-TypeIIIA	WX112	CAS-TypeIE
YC74	CAS-TypeIE	LF2	CAS-TypeIIA	WX115	CAS-TypeIIA
YC74	CAS-TypeIIC	LF2	CAS-TypeIIIA	WX115	CAS-TypeIE
YC74	CAS-TypeIIA	ATCC14931	CAS-TypeIIA	WX111	CAS-TypeIE
FJ16	CAS-TypeIE	B128	CAS-TypeIIA	WX111	CAS-TypeIIA
SD131	CAS-TypeIE	HFD1	CAS-TypeIIA	YZ156	CAS-TypeIIA
FJ181	CAS-TypeIE	KMB_613	CAS-TypeIIA	YZ156	CAS-TypeIE
CQ309	CAS-TypeIE	YN96	CAS-TypeIIA	NT153	CAS-TypeIE
XS212	CAS-TypeIE	CECT5716	CAS-TypeIE	WX121	CAS-TypeIE
XW411	CAS-TypeIE	CECT5716	CAS-TypeIC	RI_508	CAS-TypeIE
ZT591	CAS-TypeIE	DR9	CAS-TypeIIA	WX141	CAS-TypeIE
ZT25	CAS-TypeIC	DR9	CAS-TypeIE	SL1321	CAS-TypeIE
ZT251	CAS-TypeIE	DR9	CAS-TypeIIIA	Lf1	CAS-TypeIC

Foods

CQ71	CAS-TypeIIA	LMT2_75	CAS-TypeIE	LfU21	CAS-TypeIIA
GD181	CAS-TypeIIA	LMT2_75	CAS-TypeIIIA		

Figure S1: Phylogenetic analysis of cas1 and cas2 genes in the genome of *L. fermentum* from human gut and food.

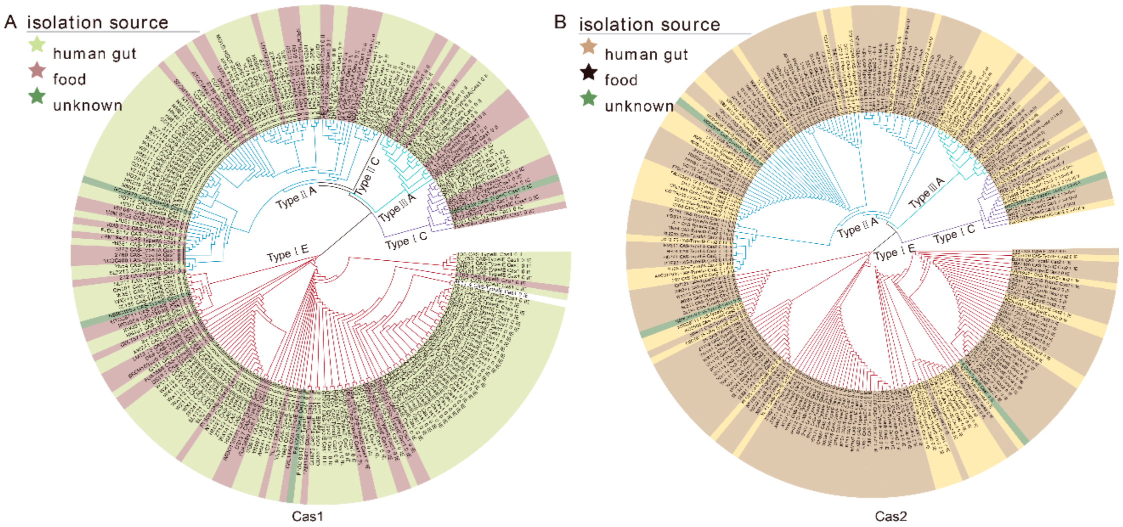

Supplement: Supplementary file 1 [file foods-11-03135-s001.zip › foods-1898652-supplementary.pdf]
